# Successful percutaneous coronary intervention with extracorporeal membrane oxygenation in a patient with acute inferior wall myocardial infarction complicated by electrical storm, cardiogenic shock, and cardiac arrest: a case report

**DOI:** 10.1186/s13019-023-02113-8

**Published:** 2023-01-24

**Authors:** Bixia Yan, Guoqi Zhang, Chaolong Huang, Zhengjiang Liu

**Affiliations:** 1 Dali University, Dali, 671000 Yunnan China; 2 Department of Cardiology, Qingyuan People’s Hospital, The Sixth Affiliated Hospital of Guangzhou Medical University, 22 Shuguang Second Road, Qingcheng, Qingyuan, 511500 Guangdong People’s Republic of China; 3 Present Address: Department of Cardiology, Youjiang Medical University for Nationalities, Affiliated Hospital of Youjiang Medical University for Nationalities, Baise, 533000 China

**Keywords:** Acute myocardial infarction, Ventricular fibrillation, Percutaneous coronary intervention, Cardiogenic shock, Extracorporeal membrane oxygenation

## Abstract

**Background:**

High-risk patients with coronary heart disease who develop acute myocardial infarction (AMI) have severe coronary lesions. If severe complications occur, such as malignant ventricular arrhythmia, cardiogenic shock, and cardiac arrest, implementation of emergency percutaneous coronary intervention (PCI) may be hindered, leading to a higher perioperative mortality rate. Extracorporeal membrane oxygenation (ECMO) can pave the way for rapid myocardial reperfusion therapy. When cardiac arrest occurs, hemodynamic support with ECMO can facilitate revascularization with PCI, which can increase the time available for further salvage and treatment and reduce intraoperative risk during PCI.

**Case presentation:**

Herein, we report a case of a 61-year-old man with AMI who suffered electrical storm of sustained malignant ventricular fibrillation, cardiogenic shock, and cardiac arrest and was successfully treated with PCI with ECMO support. During PCI, repeated aspiration and removal of the right coronary artery thrombus were performed, and blood flow was restored after right coronary artery balloon dilation. One episode of defibrillation was delivered to restore sinus rhythm. Then, stents were implanted in the distal and proximal right coronary artery lesions to achieve revascularization. After PCI with ECMO support, irreversible malignant arrhythmia returned to sinus rhythm through coronary perfusion, which prevented death following unsuccessful cardiopulmonary resuscitation. After applying active treatments, including anti-shock, mechanical ventilation, anti-inflammation, and organ support, the patient was discharged after his condition and vital signs stabilized. The patient was followed up once a week after hospital discharge, and his cardiopulmonary function recovered well.

**Conclusions:**

With ECMO support, PCI should be performed immediately in patients with inferior wall AMI complicated by electrical storm of sustained ventricular fibrillation, cardiogenic shock, and cardiac arrest to facilitate stent placement, achieve complete revascularization, restore coronary perfusion, and avoid death

## Background

When patients with acute myocardial infarction (AMI) develop severe complications, such as malignant ventricular arrhythmia and cardiogenic shock, cardiopulmonary arrest may occur, which can affect the implementation of emergency percutaneous coronary intervention (PCI), with a relatively high perioperative mortality rate. A previous study reported that extracorporeal membrane oxygenation (ECMO) combined with emergency PCI can improve the rescue success rate in patients with cardiac arrest and cardiogenic shock after AMI, with a good safety profile [1].

Here, we report a case of successful PCI with ECMO support in a patient with AMI who suffered electrical storm of sustained malignant ventricular fibrillation, cardiogenic shock, and cardiac arrest.

## Case presentation

A 61-year-old male patient was admitted to the emergency department of our hospital with sudden chest pain for 5 h. Four years prior, the patient was diagnosed with ST-segment elevation myocardial infarction (STEMI) of the inferior wall of the right ventricle on electrocardiography (ECG). He underwent emergency PCI and took oral medications for secondary prevention of coronary heart disease for a long time after the PCI procedure.

**Fig. 1:**
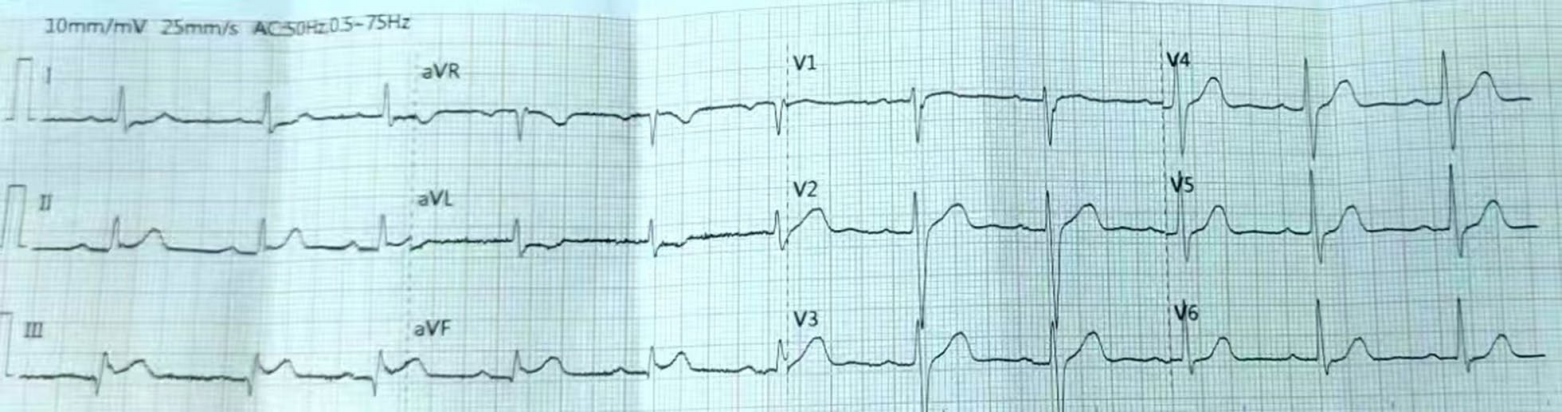
Electrocardiogram obtained at presentation to the emergency department showing acute convex upward-sloping ST-segment elevation myocardial infarction in the inferior wall in leads II, III, and aVF

On admission, the patient was confused. Breathing sounds from both lungs were coarse, and dry and moist rales were audible. The patient’s heart rate was 132 beats/min, but pendulum heart rhythms and pathological murmur were not heard over each heart valve. ECG revealed convex upward-sloping ST-segment elevation in leads II, III, and aVF, indicating acute inferior wall STEMI (Fig. 1). Routine blood tests revealed a white blood cell count of 15.18 × 10^9^/L, a neutrophil count of 9.4 × 10^9^/L, and a hemoglobin concentration of 151 g/L. Other blood parameters were within normal ranges. Cardiac enzyme tests revealed a creatine kinase concentration of 210 U/L, a creatine kinase isoenzyme concentration of 34 U/L, a lactate dehydrogenase concentration of 220 U/L, a myoglobin concentration of > 1200 μg/L, and a troponin I concentration of 0.016 μg/L.

After entering the hospital, the patient lost consciousness and developed ventricular fibrillation. Cardiopulmonary resuscitation, intubation, mechanical ventilation, and repeated electrical defibrillation were performed immediately, but ventricular fibrillation persisted. Then, 100 mg lidocaine, 2 mg metoprolol (four times), and 150 mg amiodarone were administered intravenously, followed by intravenous infusion of 300 mg amiodarone (1.5 mg/min). However, ventricular fibrillation still persisted, and the patient’s blood pressure was still 0 mmHg after 10 biphasic direct current defibrillation attempts. Emergency ECG showed cardiac arrest (with ongoing chest compressions) without pericardial effusion.

After 40 min of cardiopulmonary resuscitation, the patient was considered to have electrical storm of sustained malignant ventricular fibrillation, cardiogenic shock, and cardiac arrest due to AMI, which are indications for extracorporeal cardiopulmonary resuscitation. As the patient was comatose and experienced cardiac arrest, the use of an intra-aortic balloon pump was not considered. However, extracorporeal cardiopulmonary resuscitation was performed at the bedside. An arterial cannula (17 Fr) and a venous cannula (21 Fr) were placed in the peripheral femoral artery and femoral vein, respectively, using the Seldinger technique, allowing for drainage from the femoral vein and return to the femoral artery. The extracorporeal circulation was successfully established, and the patient was successfully connected to the ECMO machine. The ratio of blood flow to gas flow was 1:1 at a speed of 3000 rpm and a flow rate of 2.5–3.0 L. After the patient was placed on ECMO support, sinus rhythm returned, and the heart rate gradually returned to normal. The patient regained consciousness; therefore, we decided to perform revascularization under the condition of successful establishment of veno-arterial ECMO (VA-ECMO).

**Fig. 2:**
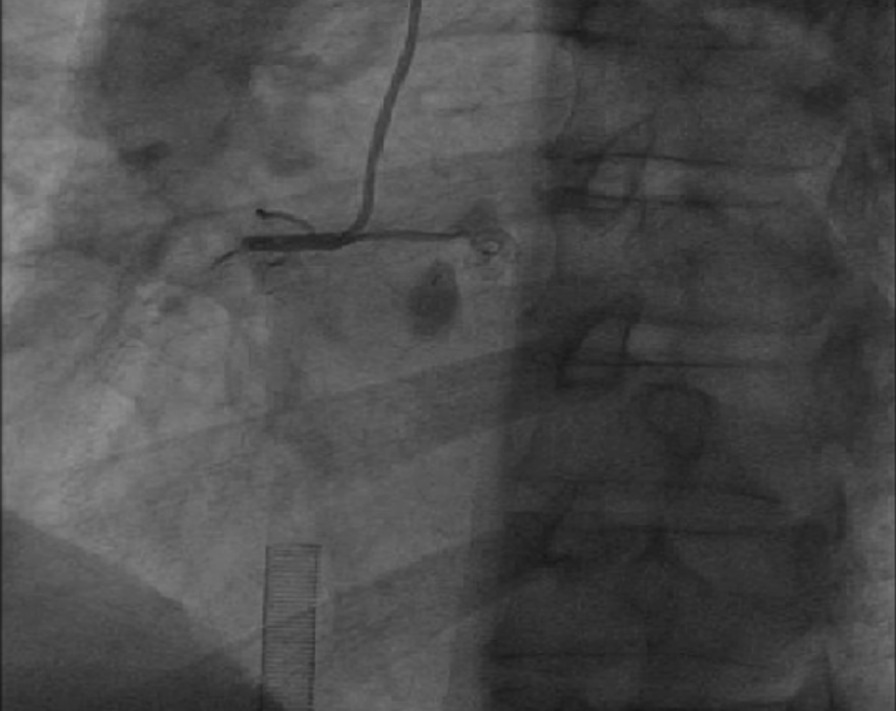
Right coronary artery angiography showing total occlusion of the proximal right coronary artery, a heavy thrombus burden at the distal right coronary artery, and a stent shadow in the middle segment of the right coronary artery, with a Thrombolysis in Myocardial Infarction flow grade of 0

**Fig. 3:**
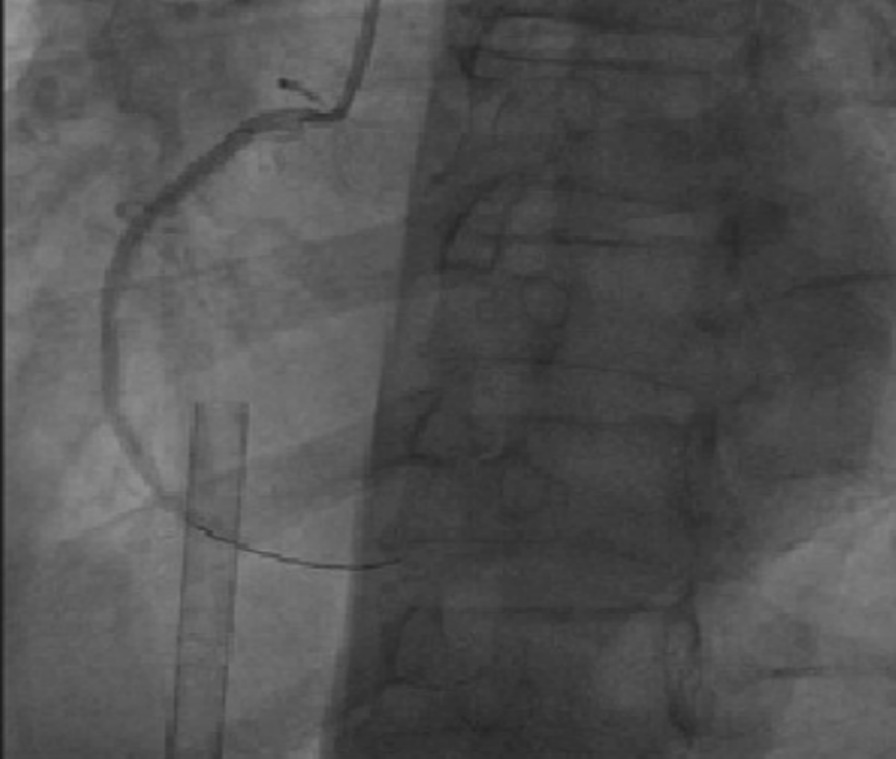
Right coronary artery angiography after primary percutaneous coronary intervention with thrombus aspiration showing that the right coronary artery was completely open. The stent shadow can be seen in the middle segment, with a Thrombolysis in Myocardial Infarction flow grade of 3

When the patient entered the interventional room, ECG again showed sustained ventricular fibrillation. Eight electrical defibrillation shocks were applied, and the patient was administered 150 mg amiodarone and 4 mg metoprolol via continuous intravenous infusion. However, ECG still showed sustained ventricular fibrillation. Thus, we decided to perform revascularization. Intraoperative angiography showed that the proximal segment of the right coronary artery was totally occluded, with a heavy thrombus burden at the distal segment. A stent shadow was visible in the middle segment of the right coronary artery, with a Thrombolysis in Myocardial Infarction flow grade of 0 (Fig. 2). During surgery, repeated thrombus aspiration was performed, and many thrombi were aspirated. After right coronary artery balloon dilation, blood flow was restored, and the patient’s heart rhythm reverted to a spontaneous heart rhythm. ECG showed ventricular escape beats and a slow heart rate of 49 beats/min. However, sustained ventricular fibrillation occurred again after the heart rate increased to 112 beats/min after intravenous injection of 0.5 mg atropine. The heart rhythm returned to sinus rhythm after delivering one electric defibrillation shock, and ventricular fibrillation did not occur. Then, one HELIOS stent (3.0 × 19 mm) was successfully implanted at the stenotic lesion in the distal right coronary artery and partially overlapped with the original stent. A GuReater stent (3.5 × 24 mm) was implanted in the proximal right coronary artery lesion and partially overlapped with the original stent (Fig. 3). The operation went smoothly.

After admission to the intensive care unit, the patient was ventilated and supported with VA-ECMO. The patient underwent continuous renal replacement therapy due to the presence of oliguria with acute renal failure. Therapies for brain protection, stress ulcer prophylaxis, glycemic control, airway care, and nutritional support, as well as therapies to reduce the systemic inflammatory response, prevent deep vein thrombosis, and maintain a stable internal environment, were provided. Thereafter, the patient’s cardiac function gradually recovered, and VA-ECMO support was withdrawn. After 7 days of treatment, echocardiography revealed an ejection fraction of 54%. Segmental left ventricular wall motion was slightly weakened, but left ventricular systolic function was normal. After 10 days of treatment, the patient was transferred from the intensive care unit to the general ward for further treatment. After 20 days of treatment, the patient’s condition and vital signs were stable, and he was discharged. The patient was followed up once a week after hospital discharge, and he did not experience any discomfort, his condition was stable, and he continued to take medication after discharge.

## Discussion

Acute STEMI is usually caused by atherosclerotic plaque rupture, which can lead to thrombus formation, partial or complete coronary artery occlusion, a severe supply–demand imbalance, and a sudden decrease in myocardial contractility. Moreover, coronary artery collapse may be imminent or may have already occurred, which is complicated by severe heart failure, malignant arrhythmia, and even cardiogenic shock [2]. Patients with AMI who are complicated by cardiac arrest have a high mortality rate. Early revascularization and restoration of coronary perfusion can reduce the risk of death, and PCI is an important tool for definitive diagnosis and revascularization in patients with coronary artery disease. During PCI, high-risk patients with complex coronary artery disease are more likely to develop severe hemodynamic disturbances followed by intraoperative adverse events, such as malignant arrhythmia, coronary non-reflow, cardiogenic shock, and cardiac arrest, resulting in adverse outcomes [3].

During PCI, ECMO does not depend on the patient’s heart rhythm and function, so it can provide a stable blood circulation and sufficient oxygen to the body and ischemic/hypoxic tissues, as well as reducing acidosis, promoting metabolite removal, promoting the recovery of the autonomic circulation, and reducing functional damage to other organs [4]. ECMO may pave the way for rapid myocardial reperfusion therapy, thereby reducing the mortality of critically ill patients with AMI and cardiogenic shock.

In the present case, the patient had severe coronary artery lesions with an acute onset, acute thrombotic occlusion of the right coronary artery, a heavy thrombus load, and ventricular fibrillation, resulting in electrical storm of sustained malignant ventricular fibrillation, cardiogenic shock, and cardiac arrest.

During the entire PCI procedure, ECG monitoring showed electrical storm of sustained malignant ventricular fibrillation after repeated administration of antiarrhythmic drugs, including amiodarone, lidocaine, and metoprolol, as well as eight electrical defibrillation attempts. When refractory ventricular fibrillation occurred, PCI was continuously performed. The guidewire was passed through the coronary artery lesion, followed by balloon dilation, repeated injection of contrast agent, aspiration, and extraction of the thrombus from the right coronary artery. Then, balloon dilation was performed again to restore blood flow. After restoration of coronary perfusion, electric defibrillation was performed to restore sinus rhythm. The stent was pushed and dilated in the distal and proximal right coronary artery lesions to achieve complete revascularization. Death following unsuccessful cardiopulmonary resuscitation due to coronary artery occlusion-induced irreversible malignant arrhythmia was averted, thus laying the foundation for subsequent treatment.

## Conclusions

The present case suggests that interventional approaches should be applied to patients with STEMI as early as possible. With ECMO circulatory support, surgeons should continue to insist that early revascularization and restoration of coronary perfusion reduce the risk of death, even if patients have electrical storm of sustained malignant ventricular fibrillation. VA-ECMO mechanical circulatory support during PCI is a safe and feasible strategy to achieve revascularization in patients with complex and high-risk coronary artery lesions.

## Data Availability

All data in the study are included in this published article.

## Abbreviations

AMI: Acute myocardial infarction
PCI: Percutaneous coronary intervention
ECMO: Extracorporeal membrane oxygenation
STEMI: ST-segment elevation myocardial infarction
ECG: Electrocardiogram
V-A ECMO: Veno-arterial ECMO
